# V180I genetic Creutzfeldt-Jakob disease with cardiac sympathetic nerve denervation masquerading as Parkinson's disease: A case report

**DOI:** 10.1097/MD.0000000000024294

**Published:** 2021-01-15

**Authors:** Hiroaki Fujita, Keitaro Ogaki, Tomohiko Shiina, Hiroki Onuma, Hirotaka Sakuramoto, Katsuya Satoh, Keisuke Suzuki

**Affiliations:** a Department of Neurology, Dokkyo Medical University, Japan; b Department of Locomotive Rehabilitation Science, Unit of Rehabilitation Sciences, Nagasaki University Graduate School of Biomedical Sciences, Japan.

**Keywords:** cardiac MIBG scintigraphy, cardiac sympathetic nerve denervation, V180I genetic Creutzfeldt-Jakob disease

## Abstract

**Rationale:**

Creutzfeldt-Jakob disease (CJD) with a point mutation of valine to isoleucine at codon 180 of the prion protein gene (V180I) is the most frequent form of genetic CJD in Japan. However, peripheral nerve involvement, especially cardiac sympathetic denervation, has not been investigated in cases with V180I genetic CJD.

We herein report a genetically confirmed case of V180I genetic CJD presenting with parkinsonism and cardiac sympathetic nerve denervation.

**Patient concerns:**

The patient was a 79-year-old Japanese woman who presented with subacute progressive gait disturbance and cognitive impairment. Clinical diagnosis of Parkinson's disease (PD) with mild cognitive impairment was initially suspected based on parkinsonism, such as bradykinesia, rigidity and tremor, and reduced accumulation of cardiac meta-iodobenzylguanidine (MIBG) scintigraphy.

**Interventions:**

Based on parkinsonism and impaired cardiac MIBG findings, levodopa/decarboxylase inhibitor was administered up to 300 mg/day; however, her symptoms were not improved.

**Outcomes:**

Her motor and cognitive function progressively deteriorated.

**Diagnosis:**

Although the patient had no family history of CJD, genetic CJD was diagnosed according to extensive hyperintensities in the bilateral cortices on diffusion-weighted magnetic resonance images, positive tau protein and 14-3-3 protein in the cerebrospinal fluid and a V180I mutation with methionine homozygosity at codon 129 by prion protein gene analysis.

**Lessons:**

We should be aware that reduced uptake of cardiac MIBG scintigraphy in patients presenting with parkinsonism cannot confirm a diagnosis of PD. CJD should be considered when patients show a rapid progressive clinical course with atypical manifestations of PD.

## 1 Introduction

Creutzfeldt-Jakob disease (CJD) is a neurodegenerative disease that can be classified as sporadic, genetic, or acquired.^[1,2]^ Inherited prion diseases, which include genetic CJD, account for 10% to 15% of prion diseases and are associated with several pathogenic mutations. CJD with a point mutation of valine to isoleucine at codon 180 of the prion protein gene (V180I) is the most frequent form of genetic CJD in Japan, which accounts for 41.2% of total genetic CJD.^[3]^ Although a few previous reports have shown the accumulation of prion protein in the peripheral nerve and sympathetic nervous system, there have been no reports about cardiac sympathetic nerve abnormalities assessed with cardiac meta-iodobenzylguanidine (MIBG) scintigraphy. We herein report a case of V180I genetic CJD presenting with cardiac sympathetic nerve denervation.

## 2 Case report

**Figure 1:**
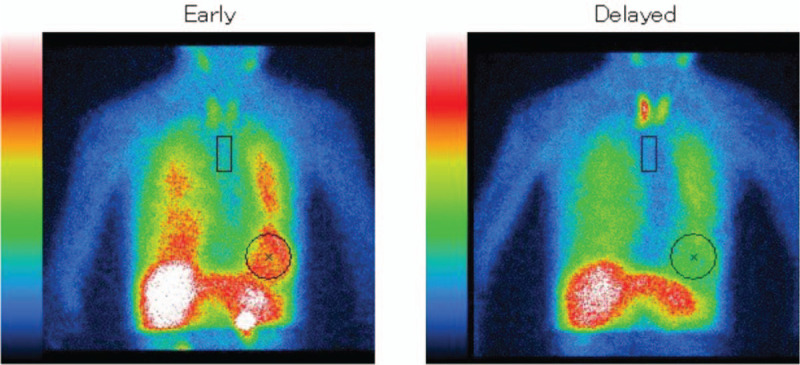
Cardiac meta-iodobenzylguanidine scintigraphy. Reduced accumulation of meta-iodobenzylguanidine indicated by the heart-to-mediastinum ratio is seen in the delayed phase.

**Figure 2:**
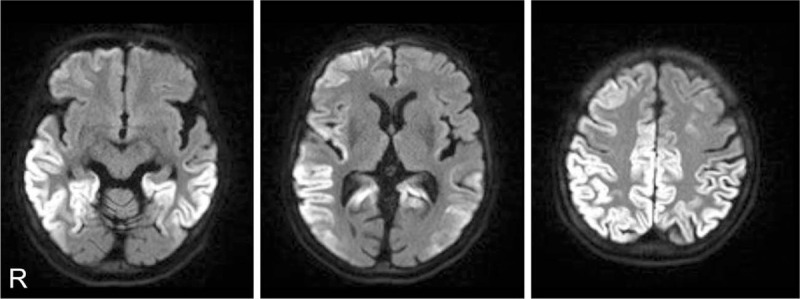
Diffusion-weighted magnetic resonance images obtained at 4 months after symptom onset. Extensive hyperintensities in the bilateral frontal, temporal and parietal cortices are seen.

A 79-year-old Japanese woman visited our department because of a progressive gait disturbance and cognitive impairment over 2 months. Subsequently, she frequently fell and had difficulty in daily activities, such as cooking and cleaning. She had no significant family history with regard to prion disease, Parkinson's disease (PD) or dementia. Neurological examination revealed bradykinesia, rigidity and kinetic tremor in her left upper extremity. Her gait involved small steps and was slow and imbalanced. PD and related disorders were initially suspected. Cardiac MIBG scintigraphy showed significantly reduced accumulation MIBG indicated by the heart-to-mediastinum ratio in the delayed phase (early phase, 2.52; delayed phase, 1.84; normal range, <2.2, based on facility reference; Fig. 1). Dopamine transporter single photon emission computed tomography (SPECT) was not performed because of the apparent parkinsonism shown on clinical examination. The score obtained on the revised Hasegawa Dementia Scale (HDS-R),^[4]^ a scale with scores ranging from 0 to 30 commonly used in Japan to assess dementia (with a score less than 21 suggesting dementia), was 23. Based on parkinsonism, cognitive testing and cardiac MIBG findings, PD with mild cognitive impairment was suspected. An oral levodopa/decarboxylase inhibitor preparation was started and increased to 300 mg/day, but parkinsonism was not improved, and her activities of daily living significantly deteriorated. Common nonmotor symptoms of PD, including episodes suggestive of rapid eye movement sleep behavior disorder, olfactory impairment and constipation, were not observed. Diffusion-weighted magnetic resonance imaging (DWI) at 4 months after symptom onset revealed hyperintense regions in the bilateral cerebral cortices (Fig. 2). By 4 months after symptom onset, cognitive impairment progressed, and she scored 10 points on the HDS-R. Electroencephalographic (EEG) studies were performed at 4 months and 5 months after symptom onset and consistently showed a diffuse slow basic pattern without periodic synchronous discharges (PSD). Cerebrospinal fluid (CSF) analysis showed normal protein levels (36 mg/dl) and cell counts (1 lymphocyte/mm^3^) and elevated neuron-specific enolase levels (NSE: 19.7 ng/ml). Semiquantitative CSF analysis revealed positive findings for 14-3-3 protein and total tau protein. A prion gene analysis using genomic DNA extracted from peripheral blood revealed a point mutation of valine to isoleucine at codon 180 (V180I mutation) with methionine homozygosity at codon 129 and glutamic acid homozygosity at codon 219. The patient was diagnosed with genetic V180I CJD at 7 months after symptom onset, by which time, she had become bedridden. Myoclonus, startle reflex or PSD on repeated EEG was not observed over the 7-month follow-up period. The patient was receiving home parenteral nutrition at the time of this report.

## 3 Discussion

We describe a genetically confirmed case of V180I CJD. The patient showed subacute progressive gait disturbance, parkinsonism, decreased activities of daily living, and cognitive decline. She did not show myoclonus or PSD on EEG. CSF showed positive results for 14-3-3 protein and total tau protein. MRI showed hyperintensity in the bilateral cerebral cortices on DWI. The clinical features of V180I CJD are as follows:^[5]^

1. Older onset age
2. Slower disease progression compared to sporadic CJD
3. A relatively low occurrence rate of neurological symptoms, such as myoclonus, cerebellar symptoms, and visual disturbances
4. Lower positive rate of brain-specific proteins, such as NSE, total tau protein, and 14-3-3 protein in CSF
5. Less PSD on EEG throughout the disease course
6. Low likelihood of family history of prion disease or dementia.

In patients with V180I with codon 129 methionine homozygosity CJD, cognitive impairment and extrapyramidal signs were observed in 100% and 53.4%, respectively. Only 5.9% had a family history of dementia, and 7.6% showed PSD during their disease course.^[5]^ The present case was consistent with these clinical features other than positive results of NSE, 14-3-3 protein, and total tau protein in CSF. We summarized previous case reports of V180I genetic CJD with parkinsonism in Table 1.^[6–8]^ All patients showed bradykinesia and 1 patient showed tremor. One patient was initially diagnosed with corticobasal degeneration. Another patient was initially diagnosed with PD and treated with levodopa without clinical efficacy. There were no patients assessed with cardiac MIBG scintigraphy.

**Table 1:** V180I genetic CJD cases with parkinsonism.

Author, year	Age, sex	Initial symptoms	Features of parkinsonism	Initial diagnosis	Family history	CSF 14–3-3 protein	CSF total tau protein	Levodopa administration	Cardiac MIBG scintigraphy
Iwasaki et al, 2011 (ref 6)	73, F	Aphasia, Apraxia, Dementia	Bradykinesia, Axial rigidity	Corticobasal degeneration	None	Elevated	Elevated	Not reported	Not reported
Hayashi et al, 2016 (ref 7)	69, F	Bradykinesia, Memory disturbance	Bradykinesia	Not reported	None	Elevated	Elevated	Not reported	Not reported
	78, F	Cognitive impairment, Tremor	Left hand tremor, Bradykinesia, Parkinsonian gait	Not reported	None	Elevated	Normal	Not reported	Not reported
	74, F	Memory disturbance, Bradykinesia	Bradykinesia	Parkinson's disease	None	Normal	Elevated	Unresponsive	Not reported
Shi et al, 2014 (ref 8)	72, F	Amnesia	Bradykinesia in lower extremities	Not reported	None	Normal	Not reported	Not reported	Not reported
Present case	79, F	Gait disturbance, Cognitive impairment	Bradykinesia, Rigidity and tremor in left upper extremity	Parkinson's disease	None	Elevated	Elevated	Unresponsive	Reduced

CSF = cerebrospinal fluid; MIBG = meta-iodobenzylguanidine.

The present case showed reduced accumulation of MIBG indicated by the heart-to-mediastinum ratio in the late phase of cardiac ^123^I-MIBG scintigraphy. MIBG is a norepinephrine analog that competes with norepinephrine for the same cellular transporter mechanisms on postganglionic adrenergic neurons. Similar to norepinephrine, MIBG is actively transported into noradrenaline granules of sympathetic nerve terminals by noradrenaline transporters.^[9]^ Recently, cardiac MIBG scintigraphy has been widely used to support the diagnosis of Lewy body disease (LBD), including PD and dementia with Lewy bodies.^[10,11]^ The reduced uptake of MIBG in LBD is thought to be the result of cardiac sympathetic denervation, and a quantitative correlation between cardiac MIBG uptake and a corresponding loss of sympathetic axons in LBD was previously indicated.^[12]^ The early phase mainly correlated with the influx of MIBG into extraneural spaces in the myocardial tissue rather than into neural components. The later phase displayed the neuronal uptake of MIBG more explicitly and correlated with the functional status of sympathetic nerve terminals. Thus, the value of the late phase is more relevant in clinical practice.^[13]^

There are some previous reports suggesting that the vagus nerve from the gastrointestinal tract, parasympathetic and sympathetic nerves, and lymphoreticular system play a central role in the pathogenesis of transmission in prion disease.^[14–19]^ Kresl et al^[20]^ reported that 5.7% of sporadic and genetic CJD cases showed prion protein immunoreactivity in the peripheral segment of the intracranial portion of the vagus nerve. Kresl et al^[20]^ also pointed out that prion protein in CJD may have similarities with alpha-synuclein deposits in PD, where the olfactory and gastrointestinal tract have been discussed as potential routes of alpha-synuclein spreading to the brain. Glatzel et al^[21]^ reported that the sympathetic nervous system was essentially involved in the process of neuroinvasion via peripheral nerves in prion disease and showed that mice with a depressed sympathetic nervous system showed a delayed development of scrapie after peripheral prion inoculation. On the other hand, mice with enhanced sympathetic innervation in lymphoid organs showed a reduction in scrapie incubation time and higher titers of prion in spleens. Haïk et al^[22]^ suggested that components of the sympathetic nervous system, including celiac and stellate ganglia, are involved in variant CJD and that the accumulation of prion protein in gut-associated sympathetic neurons may support an oral route of contamination in variant CJD patients. Khurana et al^[23]^ reported morphologic alterations in the sympathetic ganglion and vagus nerve by postmortem studies of 2 patients with spongiform encephalopathy presenting with autonomic dysfunctions. Nomura et al^[24]^ reported a sporadic CJD patient who showed various autonomic nervous system dysfunctions, including a reduced coefficient of variation in the R-R interval and abnormal diurnal blood pressure variation in the early phase. In the present case, reduced accumulation of MIBG may have reflected cardiac sympathetic nerve dysfunction affected by abnormal prion protein accumulation.

Considering the high prevalence of PD,^[25]^ the possibility that our patient was complicated with LBD, including PD, which affects the accumulation of cardiac MIBG, cannot be ruled out. Approximately 10% of people who die without a clinical diagnosis of PD display alpha-synuclein pathology in the brain and are considered incidental LBD.^[26]^ They showed mild levels of substantia nigra dopaminergic neuronal loss. In retrospective studies of unselected autopsy cohorts, abundant Lewy body pathology in the central, peripheral or enteric nervous systems was found in 24% to 55% of clinically unremarkable seniors.^[27]^ In the present case, PD was initially suspected because the patient showed bradykinesia, tremor, rigidity and reduced cardiac MIBG uptake. However, the findings of a rapid progressive course of gait disturbance and cognitive decline, severe levodopa-unresponsive parkinsonism and a lack of common nonmotor features^[10]^ in our patient did not represent the typical clinical findings and course of PD.

In conclusion, we report a case of V180I genetic CJD that showed cardiac sympathetic nerve dysfunction on cardiac MIBG scintigraphy. We should be aware that reduced uptake of cardiac MIBG scintigraphy in patients presenting with parkinsonism cannot confirm a diagnosis of PD, and CJD should be considered when patients show rapid progressive clinical course with atypical manifestation as PD. Further studies are required to confirm the involvement of the cardiac sympathetic nerve in patients with CJD.

## Acknowledgments

We would like to thank Tetsuyuki Kitamoto, MD, PhD, from the Department of Neurological Science, Tohoku University Graduate School of Medicine, Miyagi, Japan, for the genetic analysis of this patient and providing important comments on this manuscript.

## Author contributions

**Conceptualization:** Hiroaki Fujita, Keitaro Ogaki, Hiroki Onuma.

**Data curation:** Katsuya Satoh.

**Formal analysis:** Hirotaka Sakuramoto.

**Investigation:** Hiroki Onuma.

**Methodology:** Hiroaki Fujita, Keitaro Ogaki, Katsuya Satoh.

**Project administration:** Hirotaka Sakuramoto.

**Software:** Hiroki Onuma.

**Supervision:** Tomohiko Shiina, Hirotaka Sakuramoto, Katsuya Satoh, Keisuke Suzuki.

**Validation:** Tomohiko Shiina, Hirotaka Sakuramoto, Keisuke Suzuki.

**Visualization:** Keisuke Suzuki.

**Writing – original draft:** Hiroaki Fujita, Keisuke Suzuki.

**Writing – review & editing:** Keisuke Suzuki.

## Abbreviations

Abbreviations: CJD = Creutzfeldt-Jakob disease, CSF = cerebrospinal fluid, DWI = diffusion-weighted magnetic resonance imaging, HDS-R = revised Hasegawa Dementia Scale, MIBG = meta-iodobenzylguanidine, PD = Parkinson's disease, PSD = periodic synchronous discharges, SPECT = single photon emission computed tomography, V180I = valine to isoleucine at codon 180 of prion protein.
